# Unveiling the Heart of the Matter: Echocardiographic Insights into Diastolic Function and Left Ventricular and Atrial Changes in HIV Patients with Controlled Viremia

**DOI:** 10.3390/jcm13020463

**Published:** 2024-01-14

**Authors:** Magdalena Jachymek, Małgorzata Peregud-Pogorzelska, Miłosz Parczewski, Aneta Dembowska, Łukasz Wójcik, Bogusz Aksak-Wąs

**Affiliations:** 1 Department of Cardiology, Pomeranian Medical University, 70-111 Szczecin, Poland; magdajachymek@gmail.com (M.J.); malgorzata.peregud.pogorzelska@pum.edu.pl (M.P.-P.); 2 Department of Infectious, Tropical Diseases and Immune Deficiency, Pomeranian Medical University, 71-455 Szczecin, Poland; milosz.parczewski@pum.edu.pl (M.P.); anetad205@wp.pl (A.D.); 3 Department of Radiology, Pomeranian Medical University, 70-111 Szczecin, Poland; woj.luka@gmail.com

**Keywords:** cardiovascular disease, diastolic dysfunction, echocardiography, human immunodeficiency virus, left atrial enlargement, LAVI, people living with human immunodeficiency virus

## Abstract

Background: People living with human immunodeficiency virus (HIV) (PLWH) have increased risk of developing diastolic dysfunction (DD) and heart failure with preserved ejection fraction (EF). In this observational study, we evaluated DD and left ventricular hypertrophy (LVH) in PLWH receiving antiretroviral therapy (ART) with undetectable viremia. Methods: We conducted an observational study. All participants underwent transthoracic echocardiography to assess chamber size and systolic and diastolic function. Results: Most patients showed concentric remodeling without LVH. All patients had normal left ventricle systolic function (EF median 61.3%, interquartile range: 57.8–66.2). None fulfilled the DD criteria, while two patients (6%) had undetermined diastolic function. Twenty percent (*n* = 7) of patients had an enlarged left atrium (left atrium volume index [LAVI] > 34 cm^3^/m^2^). These patients had a significantly lower CD4^+^ count (771.53 ± 252.81 vs. 446.00 ± 219.02, *p* = 0.01) and higher relative wall thickness (0.50 ± 0.05 vs. 0.44 ± 0.06, *p* = 0.03). Patients without immune restoration above 500 cells/μL had significantly higher LAVI (33.92 ± 6.63 vs. 24.91 ± 7.03, *p* = 0.01). Conclusions: One-fifth of patients had left atrial enlargement associated with worse immune restoration during ART treatment. The mechanism of left atrial enlargement and its association with cardiovascular risk require further investigations.

## 1. Introduction

Globally, approximately 39 million people live with HIV and nearly 89% receive antiretroviral therapy (ART) [1]. This treatment has significantly improved the life expectancy of people living with HIV, approaching that of the general population, in areas with high ART accessibility and adherence [2]. Despite these advances, disparities in healthcare access continue to affect outcomes, underscoring the ongoing need for equitable HIV management strategies.

Cardiovascular disease (CVD) is the leading cause of death in developed countries [3]. PLWH have twice the risk for cardiovascular disease compared to uninfected individuals [4]. HIV infection may contribute to premature CVD in many ways: the chronic inflammation that persists despite suppressive ART causes endothelial dysfunction and increases pro-thrombotic activity; widespread prevalence of typical CVD risk factors; drug abuse; co-infections; and unfavorable effects of ART on the metabolic profile, promoting dyslipidemia and glucose intolerance [5,6,7]. This results in premature atherosclerosis and myocardial ischemia. On the other hand, HIV infection may also be a causative agent of heart muscle damage, causing heart failure (HF), with two possible presentations: HF with reduced ejection fraction (HFrEF) or HF with preserved ejection fraction (HFpEF). HIV-associated cardiomyopathy (HFrEF) is caused by virus-associated myocarditis and necrosis, which often overlaps with ischemia. Residual inflammation, comorbidities, such as hypertension or diabetes mellitus, and ART toxicity may also lead to increased myocardial stiffness, fibrosis, steatosis, hypertrophy, and remodeling, resulting in HFpEF [6].

HFpEF is a disease in which patients present with typical symptoms of heart failure, such as dyspnea, edema, or fatigue; however, on echocardiography, the left ventricular ejection fraction (EF) remains >50%. The diagnosis of HFpEF is based on symptoms, echocardiographic findings, and increased NT-proBNP or BNP levels. Echocardiography may reveal diastolic dysfunction (DD) with objective evidence of structural and functional abnormalities, including left ventricular hypertrophy (LVH) and concentric remodeling [8]. HIV-specific causes of DD include inflammation, particularly monocyte- and macrophage-derived fibrosis; HIV-associated microvascular dysfunction; and endothelial injury [6]. DD occurs more often in people living with HIV (PLWH) than in the general population, with DD prevalence in PLWH ranging from 8% to 25.9% [9,10,11]. As HF is more frequent and appears earlier in PLWH than in the HIV-negative population (especially HFrEF) [12], active screening for early echocardiographic changes may allow early identification and intervention.

No studies to date have focused on subclinical changes in echocardiography among PLWH, with good infection control, in a population from mid-eastern Europe, which generally has a high CVD risk [13]. This study aimed to evaluate echocardiographic changes, particularly the prevalence of DD and LVH in PLWH on ART with undetectable viremia.

## 2. Materials and Methods

### 2.1. Study Design and Population

We invited HIV-infected volunteers treated in the Department of Infectious, Tropical Diseases, and Immune Deficiency in Szczecin, Poland, to participate in an observational study that sought to assess premature, subclinical cardiovascular changes. We focused on the younger population to reduce the influence of age and the cumulative influence of long-lasting traditional risk factors. The inclusion and exclusion criteria are shown in Table 1. All study participants underwent echocardiography, coronary artery computed tomography (CACT), and carotid artery ultrasonography, performed between October 2022 and May 2023. In this study, we analyzed echocardiography results. The results of the CACT and carotid artery ultrasonography have been described elsewhere. 

The study protocol was approved by the local bioethics committee (KB-006/28/2022, 18 May 2022). The study received funding from the “Regional Initiative of Excellence” during 2019–2022, project number 002/RID/2018/19.

### 2.2. Echocardiographic Measurements

All patients underwent transthoracic echocardiography (TTE), performed by two echocardiographers, using a Philips Epiq 7C (Software Version: 3.0.3; Philips Ultrasound Inc., Bothell, WA, USA) or a GE Vivid E95 (Software Version 202; GE Vingmed Ultrasound AS, Horten, Norway).

Standard echocardiographic measurements were obtained in 2D and Doppler to assess cardiac muscle size, function, and valve sufficiency. We calculated left ventricle mass (LVM) using the following formula:

LVM (g) = 0.8 {1.04 [(LVEDD + IVSd + PWd)^3^ − LVEDD^3^]} + 0.6, (1)

where LVEDD is the left ventricular end-diastolic diameter and PWd is the posterior wall diastolic diameter [14].

We indexed the LVM to the body surface area to obtain the left ventricular mass index (LVMI). LVMI > 95 g/m^2^ in women and >115 g/m^2^ in men was diagnostic for LVH.

We calculated relative wall thickness (RWT) according to the following formula:

RWT = 2 × PWd/LVEDD,(2)

where RWT > 0.42 implied concentric remodeling when the LVMI was within the normal range (LVMI ≤ 95 g/m^2^ in women and LVMI ≤ 115 g/m^2^ in men) and concentric hypertrophy when the LVMI was increased [15].

EF was calculated using Simpson’s method, based on the four-chamber and two-chamber views. We assessed diastolic dysfunction using a scheme for preserved EF [16], that considers the following four features:

1. e′, early annular velocity of a mitral ring in tissue Doppler on the septum (e′ med) and lateral wall (e′ lat) of the left ventricle in four-chamber view (e′ med < 7 cm/s or e′ lat < 10 cm/s);
2. Average E/e′ ratio (E, peak early transmitral flow velocity) >14;
3. Maximum velocity of tricuspid regurgitation (TR V max) in Doppler >2.8 m/s;
4. Left atrial volume index (LAVI, left atrium volume measured in four-chamber and two-chamber views indexed to the body surface) >34 cm^3^/m^2^.

Meeting three or more of these criteria implied a diagnosis of DD. Meeting two criteria implied undetermined diastolic function. Meeting none or only one of the criteria implied that the diastolic function was normal [16]. 

### 2.3. Data Collection

Baseline data, including CD4^+^ count and time from the diagnosis, were obtained retrospectively from the records. Data regarding the actual viral load, CD4^+^ count, comorbidities, cholesterol fraction concentrations, body metrics, smoking status, treatment, and informed consent were collected during routine follow-up visits.

### 2.4. Statistical Analysis

Statistical analyses were performed using Statistica 13 software (StatSoft, Inc., Tulsa, OK, USA). Quantitative data are presented as mean with standard deviation (SD) or as median with interquartile range (IQR), depending on the distribution, and were compared using Student’s *t*-test or the Mann–Whitney U test. Qualitative data were compared using the chi-squared test with Fisher’s correction. Logistic regression was used to determine the odds ratio between increased LAVI and an actual CD4^+^ count of <500 cells/μL. Statistical significance was set at *p* < 0.05.

## 3. Results

The analysis included 34 non-obese patients, mostly Caucasian men, with a mean age of 41.2 years. The mean duration of infection was over 10 years (135 months). Four patients were treated because of arterial hypertension. There were no cases of atrial fibrillation. Sixty percent of patients had more than 500 CD4^+^ lymphocytes/μL, and one-third had more than 800 CD4^+^ lymphocytes, implying good immunological reconstruction (Table 2).

The results of the 2D measurements of the heart obtained during echocardiographic assessment are presented in Table 3. Most measurements were within the normal range. Most patients showed concentric remodeling with increased RWT but normal LVMI. One patient had an enlarged ascending aorta (49 mm) and a bicuspid aortic valve with moderate regurgitation. The patient was referred to the heart defects clinic for further care. All the patients had normal left ventricular systolic function.

**Figure 1:**
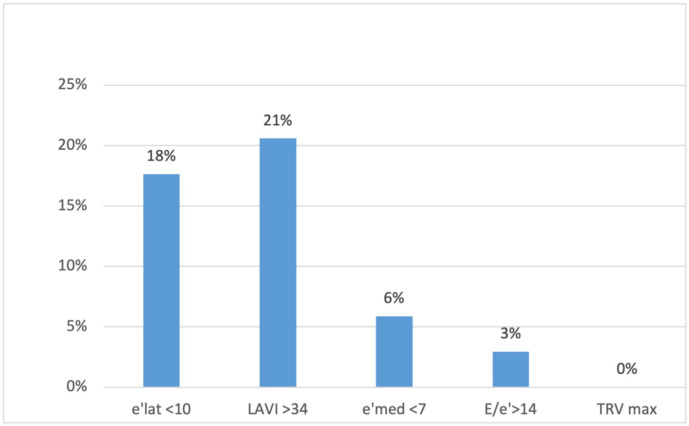
The percentage of patients presenting different diastolic dysfunction criteria on echocardiography. e′ lat, the mitral ring e′ velocity in tissue Doppler on lateral wall; LAVI, left atrial volume index; e′ med, the mitral ring e′ velocity in tissue doppler on intraventricular septum; TRV max, maximum tricuspid regurgitation velocity.

None of the patients met three or more criteria for DD, which would have allowed for a DD diagnosis. Two patients (6%) met two criteria (implying undetermined diastolic function); eleven patients (32%) met one criterion; and 21 (62%) met no criterion (implying normal diastolic function). Twenty percent (n = 7) of the patients had an enlarged left atrium, and 18% (n = 6) had decreased e′ velocity on the lateral wall (Figure 1).

**Figure 2:**
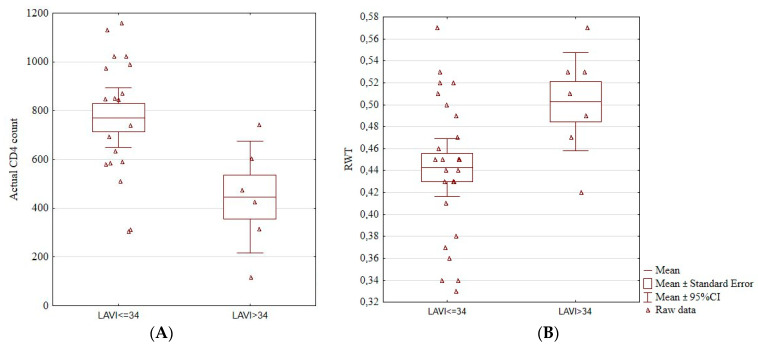
Boxplots showing a significant association between the enlarged left atrium and actual CD4^+^ cell count (**A**) and RWT (**B**). Actual CD4^+^, the number of CD4^+^ lymphocytes, LAVI, left atrium volume index, RTW, relative wall thickness, CI, confidence interval.

We found an association between an enlarged left atrium and actual CD4^+^ count. Patients with LAVI > 34 cm^3^/m^2^ had a significantly lower CD4^+^ count (771.53 ± 252.81 vs. 446.00 ± 219.02, *p* = 0.01; Figure 2A). Patients with increased LAVI also had a higher RWT (0.50 ± 0.05 vs. 0.44 ± 0.06, *p* = 0.03; Figure 2B). Among the four patients with hypertension, three had an increased LAVI (*p* = 0.02).

**Figure 3:**
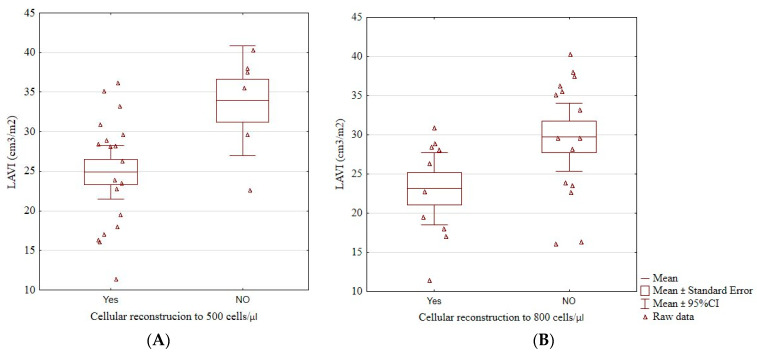
The boxplots of a significant relationship between cellular reconstruction above 500 cells/μL (**A**) and above 800 cells/μL (**B**) and LAVI. LAVI, left atrial volume index, cellular reconstruction—the reconstruction of the immune system above 500 or 800/μL CD4^+^ lymphocytes.

Patients who did not reach an immunological reconstruction above 500 cells/μL (33.92 ± 6.63 vs. 24.91 ± 7.03, *p* = 0.01; Figure 3A), and those who did not reach 800 cells/μL (29.71 ± 7.79 vs. 23.13 ± 6.42, *p* = 0.04; Figure 3B) had a significantly higher LAVI than those who did. Having more than 500 cells/μL was associated with a significantly lower risk of having LAVI > 34 cm^3^/m^2^ (odds ratio 0.06, 95% confidence interval 0.01–0.55, *p* = 0.01). No patient with LAVI > 34 cm^3^/m^2^ had more than 800 cells/μL.

We found no association between increased LAVI and age, height, BMI, LVMI, systolic blood pressure, smoking, non-HDL cholesterol concentration status, or baseline CD4 and the duration of HIV infection^+^ cell count.

There were no similar associations between low lateral e′ velocity and immune system restoration, baseline CD4^+^ and actual CD4^+^ counts, age, weight, height, BMI, LVMI, RWT, systolic blood pressure, smoking, or hypotensive treatment.

As concentric remodeling is widespread, we examined whether it was associated with the level of immune system reconstruction. Surprisingly, the relationship was quite the opposite: patients with concentric left ventricular remodeling had a significantly higher CD4^+^ cell count (776.05 ± 258.04 vs. 431.4 ± 284.04, *p* = 0.01). We found no association of concentric remodeling with baseline CD4^+^ count, reconstruction status, age, or DD criteria.

## 4. Discussion

In this study, we analyzed the incidence of LVH or left ventricular remodeling and DD among PLWH who were successfully treated with ART. We aimed to determine whether controlled HIV infection contributes to the premature development of HFpEF. Because we studied a group of relatively young (age 35–50 years), otherwise healthy PLWH, routine echocardiographic examinations revealed few abnormalities. The most common abnormality was left atrial enlargement, which was associated with worse immune restoration.

The reported prevalence of LVH in PLWH varies between studies. In our cohort, only one patient had LVH (2.94%), but 76.47% demonstrated concentric remodeling. The reported incidence of LVH in non-selected populations (including older patients with lower CD4^+^ count and already diagnosed with cardiovascular disease, such as heart failure or myocardial infarction) is higher. In an extensive retrospective analysis of TTE in the Duke HIV Echocardiography Cohort, LVH was found in 30% of the patients, while 48% of the patients had concentric remodeling [17]. In another study, LVH was found in 6.5% of transesophageal echocardiographic examinations among PLWH [18]. LVH and increased RWT are common among children with HIV/AIDS (LVH present in 66.7% cases) [19]. All these studies reported an association between low CD4^+^ cell count and LVH.

In our study, LVH occurred less frequently than previously reported, probably because of the narrow inclusion criteria. Nevertheless, only 23.53% of patients had typical heart structures without hypertrophy or remodeling. Concentric remodeling, measured using the RWT, involves small changes in left ventricular geometry. It may be an early marker of heart disease development. An elevated RWT is associated with an increased risk of death and adverse events in populations with non-anterior ST-elevation myocardial infarction [20], acute decompensated heart failure (HF) [21], HF with left bundle branch block [22], and atrial fibrillation [23]. The significance of concentric remodeling, its prognostic value, and its relationship with the CD4^+^ count in this population should be examined prospectively in a larger cohort.

The high incidence of DD among PLWH has already been described in several studies and is affected by the inclusion criteria used, such as ART status, age of the participants, or comorbidities. In our cohort, which we selected by using very narrow inclusion criteria, we had no cases of DD, and only two patients (6%) had indeterminate diastolic function. Similarly, in a prospective study of 110 PLWH on ART without a history of hypertension or diabetes, DD was found in only one case and indeterminate diastolic function was found in two cases [24]. In larger cohorts, with no restrictions on age, comorbidities, or effectiveness of ART, DD appeared more frequently. For instance, in a retrospective analysis of 228 TTEs of PLWH, definite DD was found in 8% of patients and indeterminate function was found in 38% [17]. Studies analyzing DD before ART initiation reported an even higher incidence, from 13.2% (with age as the strongest predictor of DD; 51% of patients with DD were older than 50 years) [10] to 16.5% (with age and hypertension as the strongest predictors) [25].

The most common abnormality in our study population was left atrial enlargement, which has been associated with a lower CD4^+^ count. Previous studies on DD that evaluated the relationship between LAVI and CD4^+^ have presented different results. Some studies have reported a significantly higher LAVI but no association with CD4^+^, or the association with CD4^+^ was not examined [26,27]. In the study by Hu et al. on an ART-naïve HIV-infected population, LAVI was increased compared to uninfected controls. Still, there was no correlation between LAVI and CD4^+^ count or difference in LAVI between those diagnosed with AIDS and those without AIDS [26]. In the study of Wu et al., the size of the left atrium, visualized in cardiac magnetic resonance, was associated with HIV serostatus even after adjustment for traditional risk factors (CD4^+^ count not included) [27]. One study has shown no differences in LAVI between HIV-positive and HIV-negative men, with no relationship to CD4^+^ status, but the control population was significantly older, and there was an association between age and higher LAVI [28] One study has even found a smaller LAVI in PLWH than in uninfected controls. The authors of this last study explain this difference with a higher prevalence of arterial hypertension in non-infected controls [29].

Left atrial enlargement occurs late during diastolic dysfunction and progresses with increased left atrial filling pressure. Considering the low incidence of other markers of DD, we hypothesized that the mechanism of left atrial enlargement was different. Moreover, left atrium enlargement may lead to atrial fibrillation (AF), and PLWH are at greater risk of developing this arrhythmia. A study comparing HIV patients undergoing ablation for AF to matched uninfected controls showed a different distribution of AF triggers (more common non-pulmonary vein triggers) and a higher burden of recurrence after ablation. The authors conclude that the inflammation may cause HIV-related atriopathy, leading to AF [30].

Early left ventricular damage, which is not visible on standard echocardiography, may be detected using a speckle-tracking method. Studies have shown that reduced contractility detected by speckle tracking is more likely to be found in PLWH than in HIV-negative controls [31], and that it is lower in ART-naïve groups than in treated patients [32]. Examining left atrial strain may also contribute to understanding the mechanism of left atrial enlargement.

This study had some limitations. First, the main limitation was the small sample size. However, this was partially mitigated by the in-depth echocardiographic assessment. We did not have a comparison with HIV-negative sex- and age-matched controls, which would have shown if the observed abnormalities were less prevalent in a healthy cohort. However, multiple data have already shown a greater frequency of cardiac abnormalities in HIV cohorts. Echocardiography is not a perfect technique, and the measurements and interpretation vary across readers. To reduce the possibility of bias, two independent clinicians were employed. It is impossible to distinguish all factors that may contribute to cardiovascular risk—we do not have the data on drug usage, lifestyle choices, diet, or physical activity. However, considering that the lifestyle changes over time, it is impossible to track those changes retrospectively.

## 5. Conclusions

PLWH aged <50 years without other typical risk factors for DD (hypertension, diabetes, and obesity) are probably at a low risk of developing DD. However, we found that 20% of patients had left atrial enlargement associated with worse immune restoration during ART treatment. The mechanism of left atrial enlargement and its association with cardiovascular risk need to be established in further studies. PLWH presenting with symptoms of heart failure should be diagnosed according to current guidelines, acknowledging the increased risk as compared to the HIV-negative population. Currently, the available data do not support routine screening for TTE in otherwise healthy PLWH without symptoms.

## Figures and Tables

**Table 1:** Inclusion and exclusion criteria used in this study.

**Inclusion criteria**
Age 35–50 years Confirmed infection with HIV-1 (molecular method or Western blotting test) Treatment with antiretroviral therapy Undetectable HIV-1 viral load (defined as serum HIV-RNA levels < 50 copies/mL) for at least 6 months before study enrollment
**Exclusion criteria**
Prior diagnosis of ischemic heart disease confirmed by coronary angiography Type 2 diabetes mellitus Ineffective antiretroviral therapy (HIV-1 viral load > 50 copies/mL) Low adherence to antiretroviral therapy (<80% in the last year before enrolment in the study, based on the number of packs dispensed)

HIV-1—Human Immunodeficiency Virus type 1.

**Table 2:** The descriptive characteristics of the study population.

Characteristic	Mean (±SD)/Median (IQR)/N (%)
Number of patients	34
Sex (%men)	29 (85.3%)
Age (years)	41.2 (±5.95)
Weight (kg)	78.3 (±12.99)
Height (m)	1.75 (±0.07)
BMI (kg/m^2^)	25.65 (±4.1)
Smoking	12 (35.3%)
Antihypertensive drugs	4 (11.8%)
Systolic blood pressure (mmHg)	135 (±11.4)
Time from diagnosis (months)	135 (±86.9)
Total cholesterol (mg/dL)	176 (±36.01)
High-density cholesterol (mg/dL)	46.2 (39.9–64.1)
Non-high-density cholesterol (mg/dL)	126.5 (±36.9)
Baseline CD4^+^ count (cells/μL)	406 (191–590)
Actual CD4^+^ count (cells/μL)	712 (±291.21)
Immunological reconstruction >500 cells/μL	21 (61.8%)
Immunological reconstruction >800 cells/μL	11 (32.4%)

SD, standard deviation; IQR, interquartile range; N, number of patients; BMI, body mass index.

**Table 3:** Two-dimensional echocardiographic measurements of the chamber size and related indicators.

Echocardiographic Measurement	Mean (±SD)/Median (IQR)/N (%)
Aortic diameter (mm)	29 (24–39)
Left atrium diameter (mm)	36.35 (±4.53)
IVSd (mm)	10 (8–15)
LVEDD (mm)	46 (20–58)
LVPWd (mm)	10 (8–13)
LVH (n, %)	1 (2.94%)
LVMI (g/m^2^)	90.38 (±14.99)
RWT	0.46 (±0.06)
Concentric remodeling (n, %)	26 (76.47%)
Left atrium area (cm^2^)	16.79 (±3.36)
LAVI (cm^3^/kg)	27.35 (±7.63)
LVEF (%)	61.3 (57.8–66.2)

SD, standard deviation; IQR, interquartile range; N, number of patients; IVSd, intraventricular septum diastolic diameter; LVEDD, left ventricular end-diastolic diameter; LVPWd, left ventricular posterior wall diastolic diameter; LVH, left ventricular hypertrophy; LVMI, left ventricular mass index; RWT, relative wall thickness; LAVI, left atrial volume index; LVEF, left ventricular ejection fraction.

## Data Availability

The raw data are available from the corresponding author upon reasonable request.
